# Towards more connection in drought and flood management in the transboundary Limpopo basin

**DOI:** 10.4102/jamba.v17i1.1798

**Published:** 2025-02-13

**Authors:** Anne F. Van Loon, Alessia Matanó, Sithabile Tirivarombo, Luis Artur, Rosie Day, Melanie Rohse, Syed M.T. Mustafa, Josie Geris, Simon Taylor, Zareen P. Bharucha, Farisse Chirindja, Azwihangwisi E. Nesamvuni, Anna L. Huhn, Wandile Nomquphu, Girma Y. Ebrahim, Jean-Christophe Comte

**Affiliations:** 1 Institute for Environmental Studies, Faculty of Science, Vrije Universiteit Amsterdam, Amsterdam, The Netherlands; 2 Department of Earth and Environmental Sciences, Faculty of Science, Botswana International University of Science and Technology, Palapye, Botswana; 3 Department of Economy and Agrarian Development, Eduardo Mondlane University, Maputo, Mozambique; 4 Department of Geography, Earth and Environmental Sciences, University of Birmingham, Birmingham, United Kingdom; 5 Faculty of Science and Engineering, Anglia Ruskin University, Cambridge, United Kingdom; 6 Department of Hydrology and Environmental Hydraulics Group, Faculty of Environmental Sciences, Wageningen University, Wageningen, The Netherlands; 7 School of Geosciences, University of Aberdeen, Aberdeen, United Kingdom; 8 Global Sustainability Institute, Faculty of Science and Engineering, Anglia Ruskin University, Cambridge, United Kingdom; 9 Department of Geology, Eduardo Mondlane University, Maputo, Mozambique; 10 Khanimambo Innovative Solutions, Thulamela, South Africa; 11 Department of Early Warning Systems and Anticipatory Action, World Food Programme, Johannesburg, South Africa; 12 Department of Anticipation, German Red Cross, Maputo, Mozambique; 13 Department of Water Resources and Ecosystems, Water Research Commission, Pretoria, South Africa; 14 Department of Integrated Basin and Aquifer Management, Faculty of Water, food and Ecosystems, IWMI-South Africa, South Africa; 15 Department of Integrated Basin and Aquifer Management, Faculty of Water, Food and Ecosystems, IWMI-Ethiopia, Ethiopia

**Keywords:** hydrological extremes, surface water – groundwater interactions, community resilience, water management, risk governance, forecast-based action, adaptation scenarios, transboundary semi-arid catchment

## Abstract

Improved drought and flood management in semi-arid transboundary basins requires a better understanding of the connections between dry and wet extremes, surface water and groundwater, upstream and downstream, and local communities and formal governance actors. This study describes a multi-disciplinary and mixed-methods research in the Limpopo River Basin, southern Africa. The methodology included hydrometeorological data analysis to identify drought and flood events, group discussions with 240 local community participants about drought and flood processes, impacts and preparedness, and interviews with 36 (inter)national and regional water managers and policymakers about drought and flood governance, early warning and communication. Additionally, we co-created drought and flood management scenarios through transboundary and national workshops and modelled these with an integrated surface water-groundwater model. We found that floods are crucial for aquifer recharge, providing baseflow during droughts, but also impactful for communities, who receive less training and support for floods than for droughts. Flood early warnings (if provided) are often not acted upon because of cultural values or limited resources. Drought and flood adaptation strategies were simulated to be effective, but factors like investment and maintenance costs, technical capacity and community uptake impact implementation. Furthermore, technical measures alone are inadequate to reduce community risk if underlying vulnerabilities are not addressed. Therefore, strengthening connections between communities and formal governance actors and better transboundary management of surface water and groundwater connections could yield significant benefits.

**Contribution:**

This study provides 11 distinct recommendations for managing drought and flood risk, focussing on the four connections analysed.

## Introduction

Drought and flood management are acknowledged to be often disconnected (Grobicki, MacLeod & Pischke 2015) and there are increasing calls for a better connection between them (e.g. Browder et al. 2021). However, to address this problem successfully, scientists have argued that further connections are important to consider, for example, between surface water and groundwater management (Staudinger et al. 2019), between natural and social science (Wesselink, Kooy & Warner 2017), between science and practice (Loorbach Frantzeskaki & Avelino 2017; Vogel et al. 2007) and between different levels of governance (De Stefano & Hernandez-Mora 2018; Ménard et al. 2018). In practice, however, making these connections is very challenging, especially in regions with poor data availability and limited financial and institutional resources. For transboundary aquifers and rivers (i.e. those that cross state borders), an additional challenge is that there can be large differences in data, resources and governance systems between upstream and downstream sections of the river. In this article, we address such a situation in the transboundary Limpopo River Basin (LRB) in southern Africa, where all these challenges apply. If drought and flood management in the LRB can be improved, this could have major beneficial impacts on the majority of the basin’s population (currently 18 million), especially in rural communities. In this context, we set out to investigate the potential for establishing crucial connections to develop a more integrated approach to drought and flood management. We focus on the following four connections:

- flood and drought connections;
- groundwater and surface water connections;
- upstream-downstream connections;
- connections between formal institutions and communities.

In (semi-)arid river basins such as the LRB, severe droughts and extreme floods are major concerns, but their interactions are usually not considered in modelling and management. The LRB faces both long-term water scarcity and multi-year droughts (Abiodun et al. 2018; Mazibuko, Mukwada & Moeletsi 2021), while also being highly susceptible to floods (Odiyo et al. 2019). Both droughts and floods can lead to water shortages, deteriorated water quality, crop losses and damaged water infrastructure (Ngoran, Etornam & XiongZhi 2015; Nhemachena et al. 2020). Recent studies of drought-flood interactions show that prior droughts can influence flood impacts and vice versa (Barendrecht et al. 2024), including in the LRB (Franchi et al. 2024). For instance, a 2017 flood after a multi-year drought caused significant infrastructure damage and water contamination (Chikwiramakomo et al. 2021). The sequence of these events is crucial, as multiple dry years can push systems beyond tipping points, while floods can replenish water sources (Scanlon et al. 2022). Conversely, drought adaptation measures can influence flood impacts (Ward et al. 2020). Future predictions indicate an increase in the frequency and magnitude of both extremes, particularly in southern Africa (Kusangaya et al. 2014), highlighting the importance of studying their connections.

Groundwater, with its high storage potential, could be crucial for mitigating future extremes (Geris et al. 2022; Scanlon et al. 2022), but its variability and the connections with surface water are not well understood. The response of (semi-)arid aquifers to rainfall and droughts is controlled by land surface processes and subsurface storage and flow processes. Groundwater systems connect with surface water both vertically and laterally (Maxwell & Condon 2016), especially at larger scales such as the LRB (Kapangaziwiri et al. 2021). This connectivity influences the occurrence and recovery of floods and droughts, affected by antecedent hydroclimatological events (Yang et al. 2017). Understanding these connections can help manage infrastructure and land use to mitigate floods and droughts (Geris et al. 2022). Key opportunities include improving water storage and flow management to reduce flood impacts and increase long-term water availability for future dry periods.

The spatial patterns of climate, hydrogeology and human activities within a basin create distinct upstream-downstream connections (e.g. Berhanu et al. 2016). Upstream water and land use affect downstream river and groundwater flows, for example via dams aiming to store and provide water either upstream or downstream or to prevent flooding. In the LRB, many large dams and groundwater abstractions are located upstream, related to large cities, irrigated agriculture and mining in South Africa. In addition, land use changes upstream can impact downstream water availability and flooding (Yira et al. 2016); however, research on upstream water and land use effects on downstream water users during extremes is limited (Munia et al. 2016). Some studies have shown that rainwater harvesting upstream can increase downstream water stress (Ncube et al. 2008), but dam management and water release planning can improve water sharing (Love et al. 2008). Effective management requires upstream-downstream stakeholder dialogue (Lundqvist & Falkenmark 2000), especially in transboundary basins such as the LRB.

People’s vulnerability to alternating floods and droughts is dynamic, and these events can erode communities’ coping abilities. Effective adaptation relies on good connections across spatial scales and governance types. However, there is often a disconnect between local coping mechanisms, guided by people’s experience and the urgency of the problem, and adaptation policies, informed by science and devised at (inter)national scales (SADRI 2023). Well-functioning governance is crucial for crisis management and long-term planning (Vogel & Olivier 2019), which is least available for rural communities with poor infrastructure and low coping capabilities. In the LRB, water governance varies between countries and is often short-term and reactive (Mpandeli, Nesamvuni & Maponya 2015). In South Africa, for example, local communities face challenges on account of limited communication with policymakers, and the information provided to communities is not always felt to be useful (Makaya et al. 2020).

In the previous paragraphs, we have discussed different types of connections, some more physical, and some more social. There is also a need to make connections between physical and social processes. In the LRB, there is an urgent need to develop new interdisciplinary approaches to understand the strongly interlinked physical and social systems (Mwenge Kahinda, Meissner & Engelbrecht 2016). In this study, we therefore developed an integrated basin-scale socio-hydrological approach that couples physical and social science methods. In addition, we need to make connections between science and practice. In the LRB, building resilience to extremes involves understanding the multi-faceted drivers of drought and flood vulnerability, developing suitable management strategies and strengthening the communication and knowledge flows between communities and governance across the countries (Lumbroso 2018). We, therefore, used a co-creation approach to co-design suitable and contextualised adaptation strategies and provide recommendations for policymakers.

The remainder of the article is organised as follows. First, we introduce the case study and present the mixed-methods approach integrating physical and social science approaches. Next, we discuss our findings on the current (dis)connections in dealing with drought and flooding in the Limpopo basin. This is followed by suggestions regarding which connected actions are needed to manage future droughts and floods. Finally, we provide some concluding remarks.

### Case study description

The LRB is a large transboundary river basin in southern Africa, spanning Botswana, South Africa, Zimbabwe and Mozambique (Figure 1), and its transboundary water governance institution is the Limpopo Watercourse Commission (LIMCOM). The basin has a total drainage area of 412 938 km^2^ and a total length of 1750 km (LBPTC 2010; USAID & RCSA 2002). Of the total area, 19% is located in Botswana, 45% in South Africa, 15% in Zimbabwe and 21% in Mozambique (FAO 2004). Agricultural lands and shrubs cover approximately 71% of the total basin area. The basin also includes protected areas of forest and savannah.

**FIGURE 1:**
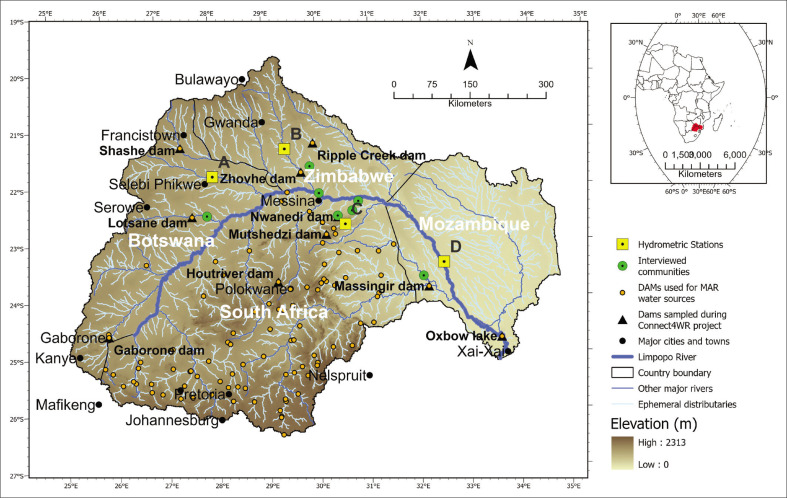
The Limpopo River Basin drainage area, including elevation, location of interviewed communities (‘Focus groups and interviews at local and regional level’ section), discharge gauging stations A–D (‘Analysis of hydro-meteorological data for the identification of drought and flood events’ section) and locations used for model scenarios (see ‘Modelling’ section).

On average across the basin, daily mean temperatures range from 20 °C in winter to 30 °C in summer. Rainfall is highly seasonal with 95% occurring between October and April. The mean annual rainfall varies from 200 mm – 400 mm in the dry areas of Botswana to 1500 mm in the southern part of the basin in South Africa and Mozambique (Mosase & Ahiablame 2018). Annual potential evaporation rates are high, 1600 mm – 2600 mm (FAO 2004). Total streamflow is 23 mm/year (Trambauer et al. 2015) and relative contributions are largely determined by the rainfall distribution, with South Africa contributing about two-thirds of river flow (LRBM 2013).

The basin has considerable groundwater resources. Groundwater storage is spatially variable, with shallow water tables and high-storage sedimentary aquifers predominantly found in downstream areas (Mozambique), as well as locally in river valley alluvial sediments spread across the basin. Lower storage aquifers with deeper water tables are found in upstream upland areas dominated by weathered-fractured basement rock aquifers (Botswana, South Africa, Zimbabwe). In addition, higher-productivity transboundary aquifers between Botswana, South Africa and Zimbabwe are locally found in karstified old sedimentary rocks and highly-fractured old volcano-sedimentary rocks (Nijsten et al. 2018).

The total LRB population currently is about 18 million and it is estimated to grow to approximately 21 million by 2040 (LRBM 2013). The largest proportion of the population is in South Africa, and the smallest in Zimbabwe. Across all four countries, there are a number of major cities and towns that heavily depend on the basin’s water (Figure 1). Major water uses in the basin include agriculture (> 50%), domestic, mining, industry, power generation and forestry. Most of the basin population relies on rainfed agriculture, fishing and livestock rearing for sustenance, while commercial farming with irrigation is also prevalent (Ingc & Fews 2003).

River flow is regulated by 20 large surface water dams (over 100 million m^3^ of capacity), located mainly in the upstream part of the basin (FAO 2004; Figure 1). Both the water stored in these dams and groundwater abstraction are used for irrigated agriculture. Highly populated areas in the upstream part of the basin abstract mostly groundwater for drinking water supply. The widespread presence of mining areas (mainly in South Africa, but also in Botswana and Zimbabwe; Maus et al. 2020) strongly affects the quantity and quality of water downstream.

The LRB is affected by both droughts and floods. Extreme droughts have severely impacted agriculture in the years 1982/83, 1991/92 and 2015/16 (Mazibuko et al. 2021). These drought events are often regional in scale with dry conditions prevalent all over southern Africa, driven by different teleconnections (Abiodun et al. 2018). The regional extent of droughts means that international actions are needed. In 1991/92, a drought disaster was prevented by coordinated regional emergency actions (Holloway 2000). In terms of floods, different types of flooding occur across the LRB: flash flooding in upstream upland regions (Chikwiramakomo et al. 2021) and widespread riverine flooding in combination with storm surges in downstream lowland regions (Lundgren & Strandh 2022). The 2000 flood reduced the annual economic growth of Mozambique from 10% to 4%, caused 800 casualties and affected almost 2 million people (Ngoran et al. 2015).

## Research methods and design

**FIGURE 2:**
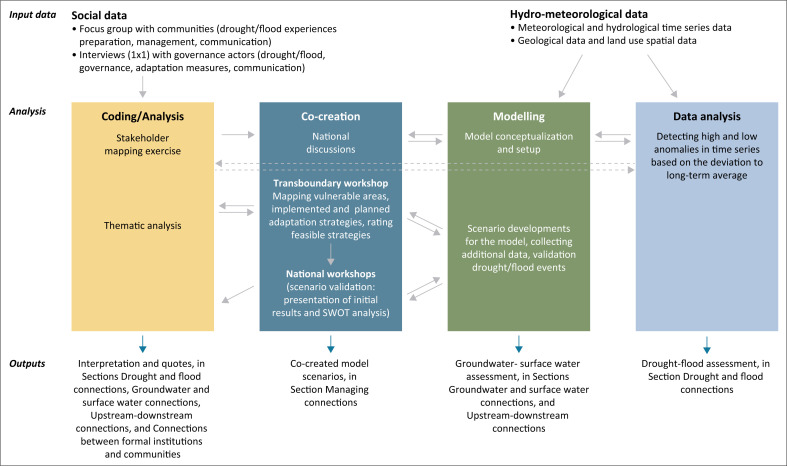
Flowchart of the mixed methods approach.

For this study, we used an interdisciplinary approach that integrated social and physical research approaches and methods. Methods involved qualitative and quantitative data collection and analysis (interviews, workshops, and time-series analysis) and integrated socio-hydrological scenario modelling. The work was carried out within the SHEAR (Science for Humanitarian Emergencies and Resilience)-funded project Connect4WaterResilience, which initially ran for 18 months from November 2018 to April 2020, but for administrative reasons and because of challenges relating to the coronavirus disease 2019 (COVID-19) pandemic (e.g. inability to travel to carry out interviews and workshops) was extended until April 2022. In this section, we will provide details of each of the methods and how we integrated them (Figure 2).

### Focus groups and interviews at local and regional level

Two sets of interviews took place in the period 2019–2021. The first set involved focus group discussions (FGDs) with community members in all four countries (Table 1). Group discussions were chosen as the method because of the social nature of knowledge formation and learning of practices, and based on previous successful use of group discussions in related work in the region (e.g. Rangecroft et al. 2018). In South Africa and Mozambique, discussions took place in three communities each, and in Botswana and Zimbabwe, in two communities each. In Mozambique, two further interviews were done with individuals because of their availability. Communities were selected to cover variation in terms of their access to water storage (for the locations, see Figure 1). Local team members led in arranging and carrying out the fieldwork. Groups were organised so that there was within-group similarity, but between-group differences (e.g. age groups), with the purpose that people would feel comfortable speaking up and sharing their views. The composition of groups was determined by local team members to reflect the most important axes of similarity and difference in the local context; age and gender were considered, but also occupation (see Table 1-A1). Recruitment happened *in situ* on field visits, with participants being given full information about the purpose of the study. In total, around 240 people participated in the group discussions across the four countries.

**TABLE 1:** Number of group discussions with communities in each of the four countries.

Country	Number of locations	Number of groups	Average number of participants per group
Botswana	2	5	7
Mozambique	3	7 + 2 individual interviews	10 (excluding individual interviews)
South Africa	3	11	7
Zimbabwe	2	6	10

The group discussions took place within the communities and were facilitated by the local team members in the local language. Lasting 90–120 min with refreshments supplied, they followed a topic guide that was common across the countries but used flexibly. Topics covered included: past experiences of floods and droughts including impacts and assistance received; current water management practices; preparation for droughts and floods, including training; forecasting methods and information use; warning systems; communication with formal governance bodies; and upstream-downstream relationships. Discussions were audio recorded and transcribed where participants were comfortable to consent, but in other cases, notes were taken during the discussion by research assistants. Translation and transcription from local languages to written English were managed within local partner institutions.

The second set of interviews were one-to-one interviews with governance actors, focussing on the local and district levels corresponding with the communities interviewed (Table 2). Following a stakeholder mapping exercise for each of the countries, governance actors for interview were identified and invited. In some cases, higher level permission was needed first. This stage coincided with the onset of the COVID-19 pandemic. This meant that access to stakeholders became very difficult as most offices were closed for several months, varying between countries and officials working in disaster management were also extremely busy. For this reason, the number and scope of interviews in most cases did not reach the initial aim, with no interviews possible in South Africa because of restrictions on officials’ activities. In total, 36 interviews took place. Interviews again followed a common protocol, used flexibly but covering the following: institutional arrangements for flood and drought governance from local to national scales; coordination between institutions; the role of informal institutions; preparation actions for floods and droughts; forecasting and response to flood and drought events; limitations on their role and actions; joined-up thinking between droughts and floods; upstream and downstream communication; communication with communities; training and support for communities; and views on community vulnerability. Interviews were carried out by local team members, largely although not exclusively online. Where possible, recordings took place, and notes and recordings were translated and transcribed into English. Both sets of interviews were analysed thematically from the English transcriptions, using NVivo software for data organisation.

**TABLE 2:** Number of interviews with governance actors in each of the four countries.

Country	Number of interviews	Organisations
Botswana	4	Ministry of Agriculture (Department of Crop Production, Department of Livestock Production), Department of Water and Sanitation, Farmers’ Association
Mozambique	10	Ministry of Water, District Health, District Education, District Economy, Recreation organisation, Water Agency, Farmers Union, Ministry of Agriculture (district and provincial), Public Construction (provincial), ARA-Sul
South Africa	-	-
Zimbabwe	22	Ministry of Water, Ministry of Environment, District and Ward officers, Water Agency, Catchment Council, provincial Met Service, provincial Development Department, Ministry of Agriculture, Agricultural Extension Services, Police, Disaster Risk Reduction (DRR) Commission, District Council senior representatives, Department of Irrigation, Ward Councillors, Provincial Administration

### (Inter)national workshops and discussions

A participatory approach comprising workshops and discussion sessions with key stakeholders on a national and international level was used to: (1) share and improve understanding of drought-flood processes, and (2) co-create management solutions to reduce impacts and increase benefits of drought-flood interactions throughout the basin.

In 2020, individual sessions were held with 17 key institutional stakeholders from national water institutes identified through snowball sampling, starting with organisations that already participated in the governance interviews (see ‘Focus groups and interviews at local and regional level’ section). The institutions involved included ARA-Sul (water agency, Mozambique), ZINWA (National Water Authority, Zimbabwe), WUC (Water Utilities Corporation, Botswana), WRC and DWS (Water Research Commission and Department of Water and Sanitation, South Africa). These sessions focussed on collecting preliminary information on current water resource management strategies to address drought and flood risk, to inform the development of management scenarios tested in the hydrological model.

Subsequently, in June 2021, a 2-day online transboundary workshop brought together 35 participants, including stakeholders from the 2020 individual sessions, representatives from the transboundary institutes LIMCOM and SADC (Southern African Development Community) and members of IWMI (International Water Management Institute) – South Africa office. The workshop aimed to present preliminary project results and discuss transboundary water resources management and resilience to floods and droughts in the LRB. Discussions in three interactive sessions identified vulnerable areas, discussed resilience strategies and considered their basin-level implications, leading to the identification of potential management scenarios.

Final national workshops in April and May 2022 presented model results for selected drought and flood management strategies, focussing on groundwater levels and river flow. Because of COVID-19 restrictions, two half-day virtual workshops were held for Zimbabwe (five external participants) and South Africa (five external participants) and a 1-day hybrid workshop for Botswana (10 external participants). Besides national water agencies, Botswana’s workshops included extension officers and Ministry of Agriculture stakeholders. The objectives of these workshops were to present the modelling assessment of current and future strategies, review implementation challenges, and identify ways forward for drought and/or flood management. Participants explored model results, added missing mitigation strategies, ranked strategies by feasibility and conducted a SWOT (strengths, weaknesses, opportunities and threats) analysis for the highest-ranked strategy.

### Modelling

We employed a collaborative basin-scale socio-hydrological modelling approach (Mustafa et al. 2024). This approach integrated in-country expert knowledge achieved through a series of regional and transboundary stakeholder workshops (see ‘(Inter)national workshops and discussions’ section) with a numerical hydrological modelling framework. A spatially distributed water balance model, WetSpass (Batelaan & De Smedt 2007), was combined with a widely used, physically based, fully distributed groundwater flow model, MODFLOW (Harbaugh 2005) using the FloPy package in Python (Bakker et al. 2016). A three-layered basin-scale groundwater flow model, allowing for a simplified representation of the basin geological variability, was initially set up based on the available, yet fragmented, hydrogeological datasets, including aquifer geometries and hydrodynamic properties. The datasets included surface and sub-surface geology and aquifer properties from SADC, the Africa Groundwater Atlas and *in situ* measurements across the LRB collected from different published articles (see Mustafa et al. 2024) and groundwater levels from the SADC Groundwater Information Portal (SADC-GIP) and SADC Groundwater Management Institute (SADC-GMI). Spatially distributed monthly groundwater recharge was simulated using the WetSpass model, based on rainfall from CHIRPS (Funk et al. 2015) and evapotranspiration (ET) from the FAO Water Productivity Open-access portal (FAO 2020). This simulated recharge was subsequently used as input for the groundwater model, and results were validated with river discharge data from the Global Runoff Data Centre (GRDC; https://grdc.bafg.de/GRDC/EN/Home/homepage_node.html) and groundwater levels from SADC. Based on the iterative feedback and inputs from the stakeholder workshops, the model conceptualisation was further improved and management scenarios were co-developed and run with the model for 50 years into the future. For more details on the model conceptualisation, inputs and parameterisation, see Mustafa et al. (2024).

### Analysis of hydro-meteorological data

For the identification of drought and flood events, we used satellite monthly precipitation data from the Multi-Source Weighted-Ensemble Precipitation (MSWEP; Beck et al. 2019) and average daily river discharge per month from the Global Streamflow Indices and Metadata (GSIM) archive (Do et al. 2018). From the GSIM dataset, four gauging stations were selected on the basis of their proximity to the communities interviewed (see Figure 1). For these stations, the monthly sums of catchment-averaged precipitation were computed.

We identified high and low anomalies in the observed hydro-meteorological time series as well as in the modelled baseflow and groundwater recharge time series. This was done by calculating the percentage deviation from the long-term average. To identify low anomalies, a variable threshold based on the long-term monthly average was employed. High anomalies were determined with a fixed threshold based on the long-term annual average.

### Integration of insights

The insights obtained from the previously described methods were integrated during several workshops involving the interdisciplinary project team. For each of the connections, at least one natural scientist and one social scientist worked together to integrate the understanding on:

- Drought and flood connections
- Groundwater and surface water connections
- Upstream-downstream connections
- Connections between formal institutions and communities.

### Ethical considerations

The study methods and protocols were scrutinised and approved by the University of Birmingham Science, Technology, Engineering and Mathematics Ethical Review Committee, reference no. ERN_18-2013A. Informed consent was obtained from participants in written form where possible, but where not possible (e.g. online interviews, low literacy), verbal consent was recorded. Participant identity was kept confidential to a sub-set of the research team and other participants in the group settings.

## Results: Connections

In this section, we combine the results obtained through the methods described earlier, drawing on both the physical and social approaches and datasets and we assess the current management of connections.

### Drought and flood connections

**FIGURE 3:**
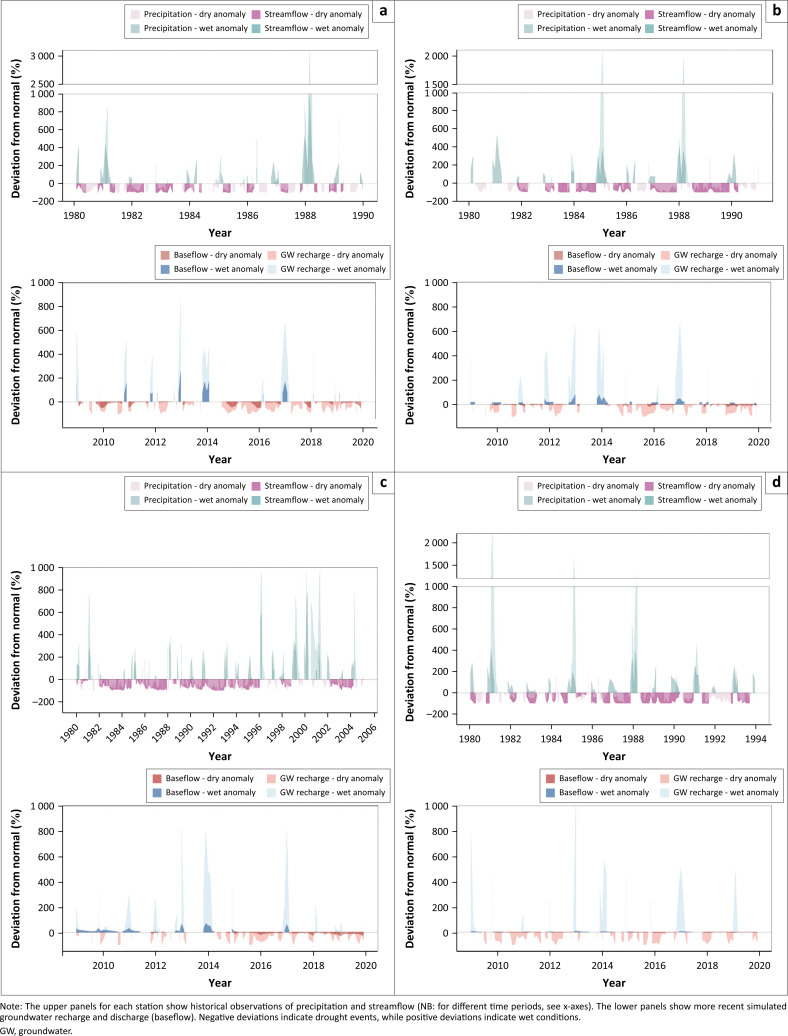
High and low deviations from normal for observed and simulated hydro-meteorological variables for four hydrometric stations in the Limpopo River Basin. The stations: (a) Tobane, Point A, Motloutse river, Botswana (11 958 km^2^); (b) Doddieburn, Point B, Umzingwani river, Zimbabwe (9 672 km^2^); (c) Tengwe, Point C, Mutaler river, South Africa (352 km^2^); and (d) Combomune, Point D, Limpopo river, Mozambique (257 367 km^2^); are situated near communities that were interviewed.

The climate of the LRB leads to strong seasonality in precipitation and streamflow, with long low (or zero) flow periods, seasonal high flow periods and extreme flood peaks in some years. Hydrological extreme events (droughts and floods) have been identified with anomaly analysis (Figure 3). Points in Botswana (A), Zimbabwe (B) and Mozambique (D) show similar extreme events with long droughts in the period 1982–1984, and in 1987 and 1989, and floods in 1981, 1985 and 1988. The droughts in the period 1982–1984 were longer and more pronounced in the upstream points A and B than in the downstream point D. And the 1985 flood was most pronounced in point B, indicating that the Zimbabwean part of the catchment contributed most to the downstream high flows (point D). South Africa (C) shows an additional drought period around 1992 and 2003 and additional floods in 1996 (ending a long drought period), 1999 and 2000. The longer record for point C allows to identify clear high-flow and low-flow periods, with low streamflow and many droughts from 1983 to 1995, and high streamflow and many floods from 1996 to 2004. Literature and community engagements confirmed severe drought in 1983–84 and widespread flooding in 2000. Model results (Figure 3) for the more recent period show prolonged droughts in 2015–16 and 2018–19 and flood peaks in 2013 and 2017, with a stronger relative deviation in recharge and a non-linear relation between recharge and discharge. The periods of extremes seem to correspond between the different points.

In the community focus groups, participants perceived droughts as worse than floods because of their longer duration, long recovery times and cascading impacts. They explained that protracted droughts impact their livelihoods and well-being by reducing their ability to grow and store food and forage, causing crop and livestock losses, and forcing them to eat less and travel further for water (Table 3 – quotes 1 and 2). In contrast, participants perceived floods as short-lived but devastating events. Some communities had been completely destroyed by floods, with the loss of human life and livestock. Both temporary and permanent relocations after flooding were reported. The hydrometeorological data analysis confirms that droughts are generally longer than floods (Figure 3). In addition, communities already face water insecurity during a normal dry season.

**TABLE 3:** Quotes from interviews and focus groups on the topic of drought and flood connections.

Quote number	Quote	Focus group or interview
1	‘This community has been affected by droughts since 2001 and the worst drought was seen in 2016 when even livestock [*mainly cattle*] didn’t resist and died. All around the community it was possible to feel the odor of the dead cattle in decomposition. There was a lot of famine because even crops in the field were also lost.’	Elderly women, focus group, Mozambique, from notes
2	‘[*In the past*] we stored our harvest for later consumption but today it is quite difficult to store anything for the future as it has been a while since it rained. For us to cope and sustain ourselves, we have to look for salary paying jobs.’	45–65 year olds, focus group, Botswana, from notes
3	**Interviewer:** ‘Have you experienced floods that followed a drought? What was that like? What was the impact of the flood? And what happened after the flood?’ **Participants:** ‘No, we have not experienced such. We normally experience long periods of drought and when we finally get water it does not instantly come as a flood.’	Women fishers, focus group, Zimbabwe, from notes
4	‘Is there any joined up thinking between droughts and floods? For example, is water from a flood stored in any way to help prevent future drought?’ (Interviewer) ‘Currently there is none, although farmers have small water storage systems. We have the biggest dam in Matabeleland South.’	Ministry of Agriculture governance actor, district level, Zimbabwe, verbatim

Community participants in all four countries did not think in terms of drought-flood cycles but considered them separately, preparing for droughts with water-saving measures and drought-resistant crops, but doing little to prepare for floods. Some (but not all) governance actors showed awareness of drought-flood management, with small dams in Botswana and Zimbabwe implemented to capture flood water for dry periods, but faced resource constraints (Table 3 – quotes 3 and 4).

Data analysis and model results (Figure 3) show three patterns: (1) drought-rich and flood-rich periods (e.g. point C 1983–1995, 1996–2004), (2) high flood peaks after long dry periods (e.g. point B 1984–85, point D 1987–88, point C 1995–96) and (3) higher baseflows after severe flooding (e.g. point B 1985, point D 1981–82, point C 2014). These patterns were not mentioned by stakeholders.

### Groundwater and surface water connections

**FIGURE 4:**
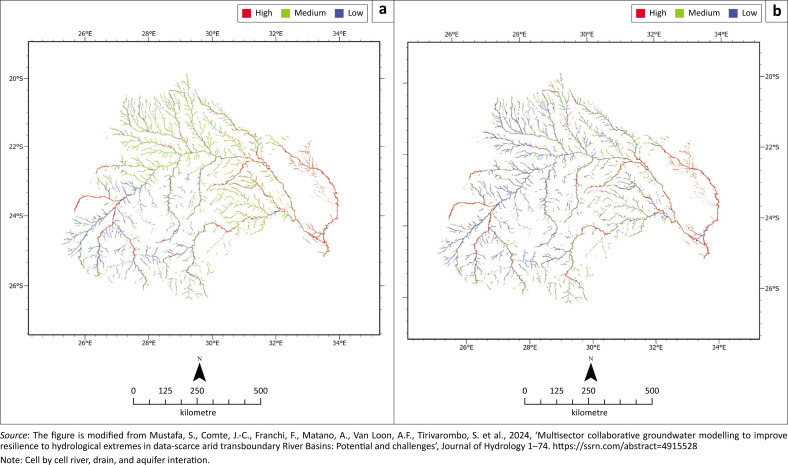
Modelled cell-by-cell groundwater-surface water interaction/exchange in the Limpopo River Basin: (a) For a wet stress period, (b) For a dry stress period. The wet stress period represents flood conditions, while the dry stress period represents drought conditions.

There is high spatial variability in groundwater-surface water interactions across the basin because of differences in climate, geology and topography (Figure 4). In the western upland and plateaus, crystalline basement and old sedimentary rocks are present. Groundwater flow and storage are low (e.g. Ebrahim, Villholth & Boulos 2019; Geris et al. 2022) and water tables are often deep (see Figure 6 in Mustafa et al. [2024]), especially in areas of higher topography. There, rivers are disconnected from groundwater and flow only during intense rainfall events. Streams are losing and groundwater recharge relies on high-intensity rainfall events and floods. Anthropogenic modifications of streams, for example, soil compaction and sand mining, cause reduced recharge and thereby increase groundwater drought.

In the upper Limpopo valleys and floodplains, alluvial deposits form productive aquifers with shallower water tables and better connectivity with surface water (Figure 4). High-intensity rainfall and floods enhance this connectivity, while meteorological drought decreases it. Alluvial aquifers are generally well connected with bedrock aquifers, but during severe droughts, water tables may fall below riverbeds and even below the alluvial deposits, drying up rivers and aquifers. Productive bedrock aquifers at low elevations in river valleys have good surface water-groundwater connections, alternating between gaining and losing conditions during dry periods and high rainfall and/or flood events (Figure 4).

In the eastern lowlands of the LRB (in Mozambique), the geology consists of younger, less consolidated sedimentary rocks with shallower water tables and higher porosity or permeability than older basement rocks (see Figure 6 in Mustafa et al. [2024]). The topography is flatter, and groundwater and surface water are well connected, making streams and rivers more permanent (Figure 4). Rivers are gaining through groundwater discharge during dry periods and losing through groundwater recharge during floods. During droughts, rivers may dry up, but groundwater still flows through alluvial aquifers (Saveca et al. 2022). In the lower Limpopo, the strong connection between surface and groundwater makes extremes appear in both water bodies simultaneously.

Groundwater and surface water are managed differently across the basin and their connection is not always recognised by water managers. From the focus groups, we found that communities rely more and more on groundwater during droughts. Some boreholes have brackish water, which worsens with increased extraction during drought and makes groundwater unsuitable for many purposes (Table 4 – quote 1).

**TABLE 4:** Quote from a focus group on the topic of groundwater and surface water connections.

Quote number	Quote	Focus group or interview
1	**Interviewer:** ‘[*A*]t the spring there is clean water?’ **Participants:** ‘[*Y*]es, there is clean water and we had to dig for it.’ **Interviewer:** ‘[*S*]o, when you drink this water, it tastes good and it doesn’t have salt in it?’ **Participants:** ‘[*S*]ome doesn’t contain salt but some does. When it contains salt, it means the water is about to dry out, when the flood stops the water was not having salt but tasting good but when water is about to run out it starts to taste salty. But we are able to use it to cook food and also give [*it to*] livestock.’	Livestock farmers, focus group, South Africa, verbatim

Stakeholder workshops revealed mixed awareness of surface and groundwater interactions. In Botswana, artificial aquifer recharge projects that harness flood water are being implemented by the government. In Zimbabwe, perceptions of joined-up management vary. Some stakeholders commented that ZINWA is responsible for surface water while local councils are responsible for groundwater, others added that ZINWA collaborates with the District Development Fund. At transboundary level, the representative of LIMCOM confirmed joined-up thinking on surface and groundwater management within their organisation, without mentioning links with other organisations.

### Upstream-downstream connections

Upstream–downstream relationships present challenges for water and disaster risk management. Downstream communities are affected by upstream socio-hydrological processes. Ideally, upstream management should mitigate downstream flooding and droughts, but often it worsens them because of water withdrawals, sand mining and reservoir operations.

In the LRB, the varied topography, soils and geology make upstream-downstream interactions complex. Rapid runoff in upstream areas with hills and consolidated subsurface increases flash flood risk, while prolonged rains cause riverine floods in the flat plains downstream, affecting settlements and agricultural lands in floodplains. Droughts develop quickly in the small streams and aquifers upstream but more slowly downstream, where communities depend on river flow from upstream during dry periods (Figure 3).

Adaptation measures such as small reservoirs in Botswana and large reservoirs like the Massingir dam in Mozambique affect downstream water flow and availability. The dams reduced medium-size flood events but did not eliminate major floods, such as the 2000 event (Tumbare 2000). This was partly because of water being kept in the reservoirs for too long and partly because of overtopping or breaking of dams (ReliefWeb 2001). Communities mentioned that the sudden release of water also caused localised flash floods, affecting water availability by washing away irrigation pumps (Table 5 – quote 1).

**TABLE 5:** Quotes from interviews and focus groups on the topic of upstream-downstream connections.

Quote number	Quote	Focus group or interview
1	‘During the 2000 flooding most of the water pumps were inundated. When there was a floodgate crack in 2008, some fuel powered water pumps were flooded. In 2013 many water pumps were inundated including Mr. XX’s. In 2013 there was early warning but it arrived late.’	Farmer interview, Mozambique, verbatim
2	‘Well, to harmonize all the stakeholders in order for them to be able to work together is very challenging because there isn’t any formal model yet. Nevertheless there is an idea to gather them and may they understand the need for a sustainable management of the water usage. They already know that up to 2025 the water available will be very low thereby there is a need for a rational water usage. Either ARA Sul, when they collect the fees, explain this to them and we also do the same. Yet a proper forum to gather them all together has never happened but it’s on the plan.’	Ministry of Agriculture governance actor, district level, Mozambique, verbatim

Upstream reservoirs can decrease water availability downstream, especially during droughts, which could increase conflict, as was mentioned by communities in Botswana. Wesselink et al. (2015) also found that in the lower Limpopo ‘previously fertile lands dried up, driving agricultural activities to zones closer to the river and to intensified irrigation’ (p. 38). This may have also increased flood exposure. In addition, unmonitored withdrawals from rivers impact downstream users and are often not considered in water management plans.

Groundwater abstraction in South Africa raises concerns about water insecurity in neighbouring countries, expressed by stakeholders during the Botswana workshops. Model results show that abstractions indeed significantly lower groundwater levels in South Africa, but this impact is relatively local (Mustafa et al. 2024). The reduced groundwater storage, however, can reduce the duration of river flow in ephemeral streams, lowering water availability downstream. This effect will be larger during a prolonged drought when abstraction is increased or with a lack of flood recharge.

Effective communication is crucial for managing connected systems. Most upstream-downstream communication is informal. LIMCOM, representing four countries, facilitates dialogue for long-term sustainable development and data collection between countries but lacks formal international agreements and an operational decision-making body for short-term actions. During droughts, early-warning information is less time-critical and informal negotiations regulate water flow. National water agencies inform farmers about water changes, but communication can fail, especially with sudden dam releases. Additional barriers to upstream-downstream communication include uncontrolled water use and local politics (Table 5 – quote 2).

### Connections between formal institutions and communities

During extremes, governments and non-governmental organisations (NGOs) send warnings and advice to communities, through TV, radio, text messaging and extension officers. Early warning systems for droughts and floods in LRB vary in accuracy and reach. Medium and long-term forecasts (2–3 months) are common for droughts, while short-term forecasts (1–2 days) are usual for floods. However, last-mile connectivity is not reliably reached in all four countries in the basin and several governance actors were frustrated with perceived community inaction, while communities expressed a lack of agency and resources.

Communities often receive warnings for both floods and droughts or no warnings at all, except for South Africa where more drought warnings are received via extension officers. Communities seemed to know better how to prepare for drought than for flood (especially in Botswana), because of their experience of recurrent drought and more drought preparedness training courses which enhanced their knowledge of coping measures. In addition, floods develop quickly, leaving little time for effective action (Table 6 – quote 1). For floods, participants only mentioned temporarily relocating and moving valuables (livestock and irrigation systems). When asked about flood preparation, there was a range of responses (Table 6 – quote 2).

**TABLE 6:** Quotes from interviews and focus groups on the topic of connections between formal institutions and communities.

Quote number	Quote	Focus group or interview
1	**Interviewer:** ‘In other words, there is nothing that you do to prepare yourselves for drought and floods [*that are*] forecast?’ **Participant:** ‘No, there was nothing at all. Heavy rainfalls can even start at night when you are least expecting it. Sometimes you would just wake up in the morning and find your house full with flood water. You wouldn’t even get a chance to see how your neighbours are doing, since you have a problem to attend to as well. You would have to remove all the flood water in the house. Sometimes you will find your water bucket flooded as well.’	Livestock farmers focus group, South Africa, verbatim
2	**Participant 1:** ‘We rely on our modern house structures as they are strong to withstand the floods.’ **Participant 2:** ‘[*W*]e move to safer places like schools, we construct trenches to allow flowing water to pass.’ **Participant 3:** ‘[*W*]e do not have any form of preparation.’	65+ year olds, focus group, Botswana, from notes
3	**Interviewer:** ‘Do you use forecasting information to anticipate droughts and / or floods?’ **Participant:** ‘Yes, we do use forecasting information to anticipate droughts/floods. We use radios that were supplied by the Meteorological Department in order to be aware of information on possible disasters. We also use Indigenous Knowledge systems from elders in the community. The information is useful and disseminated through local structures.’	District development governance actor, Zimbabwe, verbatim
4	‘There is a local [*or traditional*] early warning mechanism to protect people against floods and droughts. For instance the occurrence of the Mopane larva is an indicator of droughts approaching and when the mopane blossoms it means that there will be no droughts. There is a local fruit called Nkua that signs famine when in a season it grows big or good crop production when it grows small.’	Young people focus group, Mozambique, from notes
5	‘Two thirds of them still rely on traditional forecasting [*for droughts*] like the use of stars, indicators like the change of colours by trees e.g., *Acacia* [*Umbrella thorn*], use of insects such as beetles, excess dry and bare ground.’	18–35 year olds focus group, Botswana, from notes
6	‘People still follow beliefs from long back, that of a real man having lots of cattle. That is why even here in Matabeleland when a man died he was supposed to be buried next to the kraal or inside the kraal. Therefore there is still need to conscientise people so that they have heads [*of cattle*] they can manage during droughts … There is one problem with our communities where they do not want to desist from their old practices, people inherit norms and ideas and find it difficult to move away from them. For example during land reforms some would refuse to leave their overgrazed land for virgin land all in that his relatives were buried there hence he could not leave them and was also supposed to be buried there.’	District administration governance actor, Zimbabwe, verbatim

The uptake of forecast information varies. Low uptake can be explained by poor forecast accuracy, related to the lack of effective hydro-meteorological monitoring, limited tools for operational forecasting and the complex hydroclimate of the region (Trambauer et al. 2015). Impact-based forecasts were piloted successfully in South Africa (https://www.weathersa.co.za/home/forecastques), but the other LRB countries still need to be included. Adoption is also hindered by a lack of understanding of the highly technical information (as mentioned by farmers in Zimbabwe). Therefore, the translation of forecast information to potential actions by extension officers is very important.

The integration of indigenous knowledge (Table 6 – quotes 3–5) could enhance forecasting, increase the number of potential measures and may be more appealing to communities (Dube et al. 2024). It can also contribute to the upward flow of information from communities to policymakers. Governance actors in Zimbabwe mentioned a plan to integrate indigenous knowledge with formal forecasting information. In other countries, only community members spoke of using indigenous knowledge for forecasting; it was not mentioned by the governance actors.

Attachment to certain community traditions can also hinder the adoption of recommended interventions. To counteract this, training courses and workshops were held in all LRB countries to increase awareness of drought and flooding risks and expand knowledge on preventive actions, such as crop and fodder management. However, some actions are less acceptable to communities, such as relocating from ancestral lands, changing the grain they grow and eat, and destocking cattle (because of the high social value of herds; Table 6 – quote 6).

**FIGURE 5:**
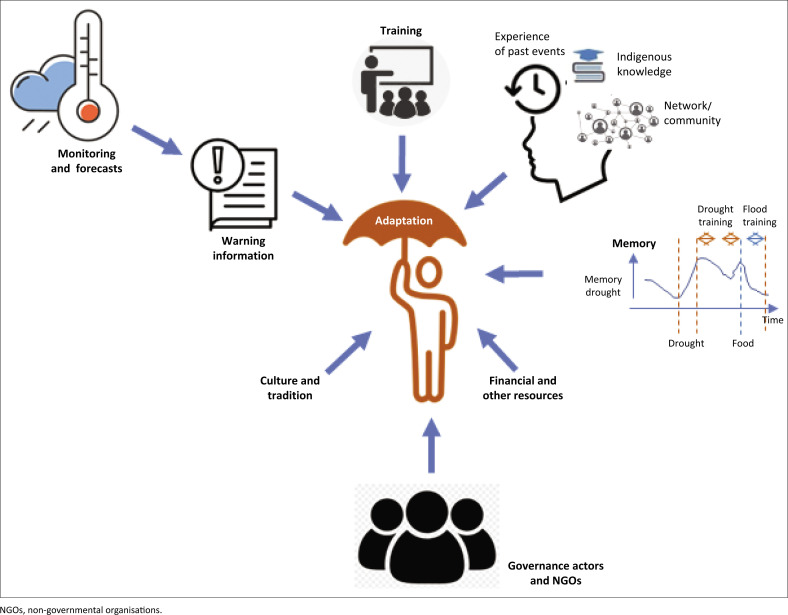
Conceptual summary of the factors affecting community adaptation to droughts and floods.

Governance actors were frustrated with communities’ apparent unwillingness to act on training, perceiving them as unprepared and expecting handouts. Communities felt a lack of agency and resources for preparation. Training effectiveness was also hindered by delays between training and flood and drought events, especially with irregular floods, leading to forgotten preparedness mechanisms. In addition, drought training was more frequent and in-depth than flood training. Factors affecting community adaptation are summarised in Figure 5.

### Managing connections

In this section, we discuss adaptation and coping measures for drought and flood implemented by communities and formal governance actors in LRB and model the effect of several of these measures in a scenario analysis. To prepare for and respond to drought and flood events, communities and governments adopt various short- and long-term actions. Comparing countries shows no large differences in drought and flood measures (Table 2-A1 and Table 3-A1), except for South Africa where communities apparently prepare less and receive less training.

Generally, communities engage in low-cost drought preparedness measures such as planting drought-tolerant crops, storing crops ahead of forecasted droughts and attending government training. For drought response, they rely on government aid, digging riverbeds for water and increasing groundwater abstraction. Diversification of livelihoods, mainly through migration to cities, is also a response to drought. For flooding, communities strengthen structures, move irrigation equipment or relocate to higher ground. They rely on humanitarian aid during floods, although it is more sporadic than during droughts. There was no consensus within or between communities on strategies, and some could not be implemented because of the lack of funds. Government stakeholders noted the need for information on the effectiveness of community strategies.

From the workshops, we found that government strategies differ from community measures. To address potential improved future management, with diverse government actors, we co-developed several drought and/or flood management scenarios and modelled these using the socio-hydrological model (see ‘Modelling’ section and Mustafa et al. 2024). The developed scenarios were: deforestation, afforestation, and managed aquifer recharge (MAR) using injection wells near major reservoirs, infiltration via small in-stream reservoirs (e.g. sand dams), and rainwater harvesting and infiltration through local ponds (with evaporation) and local wells (without evaporation). The model simulated both constant abstraction and an increase in groundwater abstraction in the future.

**FIGURE 6:**
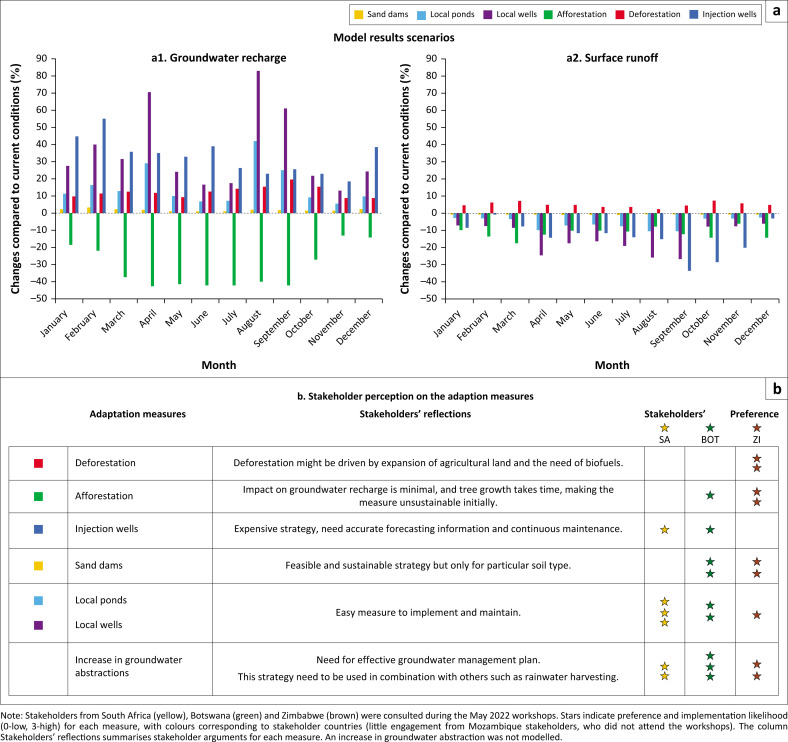
Assessment of drought-flood adaptation measures according to model analysis and stakeholder perception. a) Bar charts showing changes in basin-average gridded groundwater recharge (panel a1) and surface runoff (panel a2) over time for land use and land cover change measures and managed aquifer recharge techniques according to model results. (b) Stakeholder perception of the effectiveness and likelihood of implementing the drought-flood measures investigated.

Model results show that, from a technical-hydrological point of view, a combination of afforestation and MAR would be optimal for coping with drought-flood cycles. Afforestation limits surface runoff and flash floods (on average surface runoff decreases by 11%; Figure 6-a2). Managed aquifer recharge via different techniques can use floodwater to recharge aquifers (on average aquifer recharge increases by 24%; Figure 6-a1), increasing groundwater availability during droughts. This can offset increased groundwater abstraction resulting from afforestation, increasing population and irrigated agriculture.

The largest simulated increases in aquifer recharge were obtained through MAR using rainwater harvesting via local ponds or local wells (Figure 6-a1), which decreases drought severity. These measures also reduce surface runoff and flood severity (Figure 6-a2). Managed aquifer recharge injection wells near existing reservoirs also increase groundwater levels, but the impact is more local (5 km – 50 km) and limited by the number of reservoirs connected to boreholes. This also can decrease streamflow peaks as water is injected when reservoirs reach maximum capacity, thereby potentially reducing downstream flash floods from overtopping reservoirs (see ‘Upstream-downstream connections’ section). More detailed model results can be found in Mustafa et al. (2024).

Contextual conditions, however, affect the choice of optimal drought and/or flood management measures, as indicated in the stakeholders’ workshops (Figure 6b and Figure 1-A1) and compromises are needed between geophysical characteristics and socio-economic conditions. Important factors are investment costs, technical capacity and community uptake. For example, although all four countries have groundwater and surface water management plans (some more than others), regulations are rarely enforced, leading to ineffective implementation of measures. In all country workshops, participants noted that the lack of an integrated groundwater management plan hindered any intervention in the basin. Excessive abstraction and water pollution also reduce the effectiveness of drought and flood measures. There are many criticisms of afforestation, for example, that trees have high water use, often exotic species are planted in monoculture, local communities do not benefit from the plantation and it can alter climatic patterns potentially increasing drought elsewhere (Gautier et al. 2015; Naik & Abiodun 2016); therefore, these projects should be implemented with care (Reisman-Berman, Keasar & Tel-Zur 2019).

Mitigation and preparedness strategies must consider community livelihoods and traditional practices. In Zimbabwe, sand dams as pilot projects have enhanced community livelihoods and ecosystem services (Lazurko et al. 2024), unlike other strategies that ignore community-livestock interdependence. Sand dams increased water availability, supporting current livelihoods, providing new opportunities and enhancing nutritional security via fresh vegetables. However, downstream effects must be considered, as decreased river flow during dry periods could increase tensions between communities (see ‘Upstream-downstream connections’ section). In South Africa, stakeholders noted that local solutions work better as they can be quickly implemented without bureaucracy and communities can easily maintain them.

## Recommendations: Towards connected action

In this section, we provide our recommendations for managing the connections between floods and droughts, between surface water and groundwater, between upstream and downstream, and between local communities and formal governance actors.

### Flood and drought impacts and preparedness

The impacts of floods and drought on communities are high. Communities have coping strategies but often lack preparation capacity. Forecasting is available, but last-mile connectivity can be improved and various factors (training, memory, resources and traditions) affect early-warning responses.

### Flood benefits

Floods can recharge aquifers and enhance water security during dry periods. Communities could maximise these benefits through actions related to rainwater harvesting and governments can implement technical measures such as infiltration wells near reservoirs. Infrastructure must, however, be flood-proof and not harm downstream areas.

### Integrated training

More comprehensive training for communities and extension officers on drought and flood preparedness is needed. Drought training should address the unintended effect of drought adaptation on flood risks, and flood preparedness should focus on protecting drought adaptation infrastructure.

### Flood preparedness

Improved flood preparedness would allow communities to benefit from wet periods and reduce damage, for example, with measures that reduce surface runoff and increase groundwater infiltration. Better training, information flows and early-warning systems are crucial, especially for downstream communities and in relation to sudden reservoir releases.

### Transboundary treaty and forecast-based action

A treaty on water distribution during dry periods is recommended to prevent downstream issues and conflicts. In addition, impact-based forecasting and coordinated anticipatory actions, supported by pre-allocated financing, could be implemented. Limpopo Watercourse Commission (LIMCOM) and the South Africa Weather Service can play key roles.

### Upstream measures

Decreases in infiltration capacity (e.g. through sand mining) should be prevented and measures to increase infiltration (MAR) should be implemented. To maintain ecosystem integrity, land use activities should always fall within the regulated buffer zones. Afforestation should be done carefully, learning from established good and poor practices, to avoid negative impacts.

### Subsurface water storage

Subsurface storage (increased with MAR) can be used more intensively; especially in Botswana and Zimbabwe, there is room for increased groundwater use. However, salinity and transboundary effects must be considered. Basin-scale surface water-groundwater models, groundwater management plans and a licensing process for groundwater abstractions are needed.

### Avoid maladaptive consequences

Stakeholders should be aware of the potential negative impacts of actions on downstream communities and other sectors. In addition, non-structural measures to increase resilience should be developed and implemented (Krysanova et al. 2008).

### Community benefits

It is crucial that communities with fragile livelihoods existence benefit from measures. Government and NGO actors should change their perspective from focussing on hazards to underlying vulnerabilities and lack of resources of communities, which undermines their agency to prepare well for floods and droughts (Lundgren & Strandh 2022). Low-cost local measures such as rainwater harvesting should therefore be promoted and supported.

### Collaborative approach

Government, NGOs and communities must work together with sufficient resources for the implementation of measures and their maintenance, especially during and after extreme events (Bahta 2021). This helps to move from crisis response to proactive management (Vogel & Olivier 2019).

### Role of intermediaries

Extension officers and River Basin Organisations are vital links between communities and district or regional government. They manage two-way information flows, can translate policies into practices and share community concerns and needs with government agencies (Makaya et al. 2020; Wheeler et al. 2017).

## Conclusion

Our research shows the complexities of the physical and social connections that play a role in flood and drought management in a semi-arid transboundary basin characterised by low financial and institutional resources and a weak connection between formal governance actors and communities. We also find that training and early warnings are provided and that communities engage in several low-cost measures for droughts and floods, both preparatory and responsive. However, identified optimal strategies can often not be implemented, warnings are not always acted upon because of a combination of the lack of resources and cultural reasons, and proactive management plans and enforced licences are lacking. There is ample room for improvement in flood and drought management, taking into account the connectivities between droughts and floods, between surface water and groundwater, between upstream and downstream, and between formal governance actors and communities. This would allow for all complexities and indigenous knowledge to be included and would avoid maladaptations.

We focussed on the LRB because it encapsulates a diversity of physical and socio-economic characteristics requiring investigation of the (dis)connections, both physically (flood-drought, upstream-downstream and surface water-groundwater) and socially (contrasting politics, cultures and economies). This makes our assessment relevant across multiple geographical and socio-political contexts in sub-Saharan Africa and beyond. Our recommendations (see ‘Recommendations: Towards connected action’ section) can be used to develop approaches to resilience to droughts and floods in other (transboundary) river basins. In addition, the methodology (see Figure 2) can be replicated in other basins.

## Figures and Tables

**FIGURE 1-A1:**
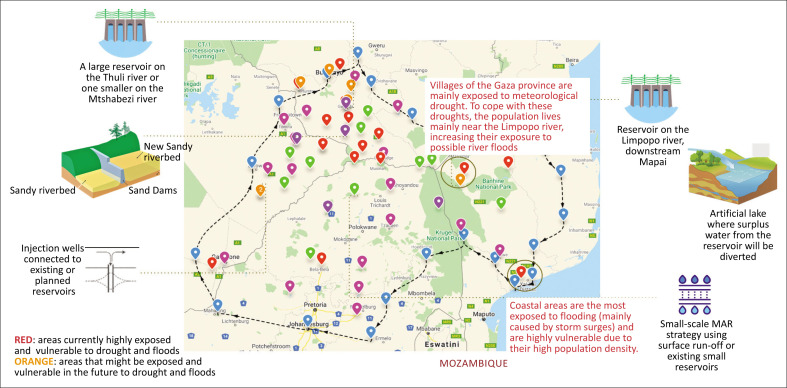
The stakeholders’ workshops.

## Appendix 1

**TABLE 1-A1:** Composition of focus groups and interviews at local and regional levels.

Country	Group profile	Number of participants	Notes
**Botswana**			
Maunatlala	18–35	4	-
Maunatlala	35–45	5	-
Not stated, presumably Maunatlala	45–65	9	-
Not stated, presumably Maunatlala	66+	9	-
Maunatlala	Non-dam users	At least 3	‘Farmers and Pastor’
**Mozambique**			
Bingo	Men	12	Aged between 30s and 80s
Bingo	Women	10	-
Bingo	Youths	Not stated	-
Massingir	Farmer (single interview)	1	-
Massingir	Fisherman (single interview)	1	-
Mukatine	Older men	5	Ages not given
Mukatine	Older women	11	Aged between 40 and 55 but they do not all know their age
Mukatine	Young men	9	Ages not given
Mukatine	Young women	11	Aged between 18 and 31
**South Africa**			
Mukovhabale	Livestock farmers	8	-
Mukovhabale	Arable farmers	10	-
Mukovhabale	Domestic water users	8	-
Mukovhabale	Land care	7	-
Gumbu	Livestock farmers	6	-
Gumbu	Arable farmers	6	-
Gumbu	Domestic water users and seed bank initiative	6	-
Tshipise	Livestock farmers	6	-
Tshipise	Arable farmers	6	-
Tshipise	Domestic water users	8	-
Tshipise	Youths	6	-
**Zimbabwe**			
Ndambe (ward 7)	Fisherwomen	12	-
Ndambe (ward 7)	Fishermen	11	-
Ndambe (ward 7)	Other men - farmers/other occupation	8	-
Mazunga (ward 14)	Women livestock farmers	9	-
Mazunga (ward 14)	Men farmers/other	11	-
Mazunga (ward 14)	Youth (all in 20s)	7	-

**TABLE 2-A1:** Drought adaptation and response actions by communities for each country within the Limpopo basin (based on community focus groups).

Country	Preparatory actions – drought	Responsive actions – drought
South Africa	- Sell cattle and goats after drought warning – reluctant, and not universal - Ask the extension officer for advice, and apply to the government for assistance - Pray - Gather leaves to feed cattle	- Take up government aid (livestock feed, food parcels) but this is insufficient and patchy - Children get school meals - Reduce food consumption to one meal a day - Cut branches to feed livestock
Botswana	- Preserve food in advance - Grow grain sorghum which can be preserved for livestock and people - Store harvest and advance buy animal feed, if able - Store water in storage tanks (some) - Use ploughing methods recommended by the Ministry of Agriculture - Take up government training on livestock management in drought - Some do not prepare, as don’t feel they are warned/do not feel able to store anything - Look for paid work	- Blast to access groundwater - Dig water trenches near the riverbank to store water - Build ridges to channel water - Collect rainwater - Most received government aid: food, water and animal fodder; some from the United Nations
Zimbabwe	- Buy animal feed, if affordable - Construct storerooms to store animal feed for the dry period - From training, plant drought-resistant crops - Re-use grey water to water gardens - Take-up training on crop and livestock management from extension officers - Construct contour ridges - Some limited use of training to dig infiltration trenches - Reduce siltation to ensure they don’t lose water - Diversify income (e.g. selling handicrafts)	- Dig wells along the river - Limit fishing in the dam (following ZINWA advice) - Try to sell livestock, but suffer losses - Buy animal fodder from South Africa - Take temporary paid work to buy food and fodder - Some receive food aid, often from overseas agencies - Children get school meals, provided by aid agencies
Mozambique	- Perform traditional rituals (older men only, and this is dying out, which they see as the cause of recent droughts) - From training, plant drought resistant crops – cassava and maize – but cassava recently hit by disease - Some have taken up NGO training in hay production to feed livestock	- Dig for water on the river bed (women) - Reduce food consumption to one meal a day - Eat wild plants - Move cattle to where there is more water and pasture - Try to sell cattle (reluctant) - Cultivate closer to the river - Reduce water usage in the home – for bathing, dishwashing - Children receive school meals, provided by aid agencies - Receive food and money from aid programmes - Migrate to South Africa for temporary paid work (men)

ZINWA, National Water Authority, Zimbabwe; NGO, non-governmental organisation.

**TABLE 3-A1:** Flood adaptation and response actions at the community level for each country within the Limpopo basin (based on community focus groups).

Country	Preparatory actions – flooding	Responsive actions – flooding
South Africa	- Nothing	- Receive government food parcels and tents where available – but minimal and not universally received - Farmers come together to assess irrigation damage and replacement - In 2014 specifically, managed to collect some rainwater that was treated and used for livestock
Botswana	- Build stronger house structures - Build on higher ground - Little to no other preparation	- Temporarily evacuate to higher ground if necessary - Receive assistance with evacuation, shelter and emergency food from government and foreign agencies
Zimbabwe	- Build stronger houses - Build away from waterways - On receiving a warning, may relocate to higher ground – but note landslide risk - On receiving a warning, keep people away from rivers and keep children home from school	- Move to higher ground if becomes necessary - Move livestock - Have received some help from donors – food and clothes, but little - Traditional leaders and local businessmen support the community - Community comes together to rebuild houses
Mozambique	- Following the bad 2000 flood, have rebuilt on higher ground - On receiving a warning, move irrigation equipment - No other preparation, even though they receive warnings	- Move to safe ground, if becomes necessary - Climb trees in an emergency - Have sometimes received humanitarian aid from government and external agencies, but not more recently - Return and rebuild when able
